# The Effect of Freeze-Dried Cherry Pomace and Red Potato Pulp on the Content of Bioactive Substances in Pasta

**DOI:** 10.3390/ijms26136020

**Published:** 2025-06-23

**Authors:** Dorota Gumul, Wiktor Berski, Eva Ivanišová, Joanna Oracz, Marek Kruczek

**Affiliations:** 1 Department of Carbohydrate Technology and Cereal Processing, Faculty of Food Technology, University of Agriculture in Krakow, Balicka 122 Str., 30-149 Krakow, Poland; rrberski@cyf-kr.edu.pl (W.B.); marek.kruczek@urk.edu.pl (M.K.); 2 Institute of Food Sciences, Faculty of Biotechnology and Food Sciences, Slovak University of Agriculture in Nitra, Trieda Andreja Hlinku 2 Str., 94976 Nitra, Slovakia; eva.ivanisova@uniag.sk; 3 Institute of Food Technology and Analysis, Faculty of Biotechnology and Food Sciences, Lodz University of Technology, Stefanowskiego 116 Str., 90-924 Lodz, Poland; joanna.oracz@p.lodz.pl

**Keywords:** fortified wheat pasta, antioxidant potential, polyphenols profile, phytosterols

## Abstract

Pasta, due to its convenience, follows bread as the most common cereal product in the human diet. Typical wheat pasta is a high-energy product, since it contains a large amount of starch; at the same time, it is characterized by a low content of health-promoting ingredients, such as dietary fiber, minerals, vitamins, and polyphenols. Food industry by-products, or even waste, can be applied as a source of many bioactive substances, thus enriching pasta with bioactive ingredients. Two by-products, Cherry Pomace (CP) and Red Potato Pulp (RPP) were applied as health-promoting supplements for wheat pasta, at three levels (10, 20, and 30%). The antioxidant potential of the resulting pasta was examined (by DPPH, ABTS, FRAP, and FOMO methods), and the antioxidant’s content was also tested. The amount of polyphenols determined by HPLC was higher in the case of CP than in RPP, and the main ones were 5-O-Caffeoylquinic acid and Cyanidin 3-O-rutinoside in CP, whereas for RPP it was Pelargonidin 3-(4‴-p-coumaroylrutinoside)-5-glucoside. Fortified pasta samples were characterized by a higher content of total polyphenols and phenolic acids, flavonoids, flavanols, and anthocyanins. In pasta with a share of CP, some polyphenols were unstable during pasta production. Pasta with a share of CP was characterized by very high antioxidant activity due to a high level of phenolic acids and anthocyanins acting synergistically. It was also characterized by a higher content of phytosterols. A 30% addition of CP into pasta is considered the most beneficial in terms of increasing the health-promoting properties of such a product.

## 1. Introduction

Pasta, alongside bread, is the most common cereal product in the human diet. Pasta is popular because it is easy and quick to prepare, durable and quickly satisfies hunger [1,2,3]. Although bread is the most basic starchy product in Poland, pasta is gaining an increasing number of supporters. Unfortunately, pasta is a high-energy product because it usually contains a large amount of starch, and has a low concentration of health-promoting ingredients, such as dietary fiber, minerals, vitamins, and phenolic compounds [4]. Nevertheless, these products have a low glycemic index among high-starch products. Therefore, they can be consumed both by healthy persons, as well as by people suffering from various diseases such as diabetes, atherosclerosis, gout, or some cardiovascular diseases [5].

Many additives can be applied to make pasta more attractive. Therefore, many scientific studies have focused on increasing the health-promoting value of pasta by adding functional ingredients, which will contribute to reducing the risk of many diseases [6]. The addition of protein, antioxidants, dietary fiber, and omega-3 fatty acids to pasta will result in gaining the title of functional food [7]. The benefits of consuming such pasta include its anti-inflammatory [8], antioxidant [9], and anti-diabetic properties [5,10,11,12]. In order to make pasta more attractive and increase its health-promoting properties at a much lower cost, it is necessary to take into consideration that waste or by-products can be a source of many bioactive substances and thus enrich pasta with bioactive ingredients.

Modern trends in the food industry and agro-food research have set the priority of reducing waste from fruit and vegetable processing by improving recipes and food production methods. Zero waste technology is one of the 17 sustainable development goals adopted by all member states of the United Nations [13]. Additionally, by-products and agro-food residues, through appropriate extraction or microbiological stabilization methods, e.g., by freeze-drying, will make it possible to utilize these raw materials in the production of innovative products. This is an invaluable opportunity to contribute to the sustainable development of the sector, also bringing economic and social benefits to the population, and saving the environment [4,14].

Therefore, in this research, two by-products, Cherry Pomace (CP) and Red Potato Pulp (RPP), were applied, and their influence on pasta’s health-promoting properties was investigated. A lot of cherries are collected in Poland every year, which in turn generates a very large amount of wasted raw materials in the form of pomace. CP is a common residue from the production of nectars, soft drinks, jams, and juices. It is an invaluable source of fiber components, vitamins, and minerals, but above all, it is a source of polyphenols from the group of both anthocyanins and hydroxycinnamic acids, quercetin, and procyanidins [15]. In turn, RPP is created as a result of starch isolation, and its amount oscillates around 1.36 tons for every ton of starch produced [16]. This pulp contains a lot of fiber, which includes polyphenols, as well as a very large amount of polyphenolic compounds, including anthocyanins stabilized by phenolic acids [16]. Therefore, in this research, an attempt was undertaken to produce wheat pasta with a share of CP and RPP.

This research aimed to investigate the influence of different levels of two by-products: Cherry Pomace (CP) and Red Potato Pulp (RPP) on the quantitative and quality profile of polyphenols and phytosterols in wheat pasta (prior to chemical analyses, the pasta samples were cooked and freeze-dried). Then the antioxidant potential of the resulting pasta was examined using four methods (DPPH, ABTS, FRAP, and FOMO). Moreover, this work aimed to answer the question of which of the additives is the best for producing pasta with a high antioxidant potential, which will result in the health-promoting properties of such pasta.

## 2. Results and Discussion

### 2.1. CP and RPP Characteristics

**Figure 1:**
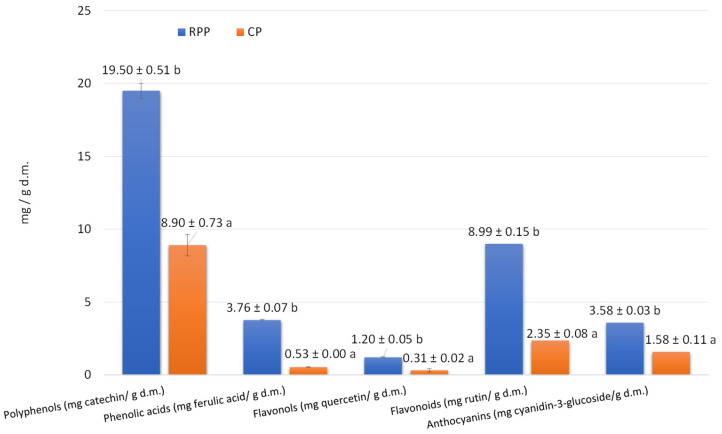
Content of bioactive compounds in Cherry Pomace (CP) and Red Potato Pulp (RPP). Note: a,b Superscript letters indicate statistically significant differences (α = 0.05).

When comparing the two types of additives, i.e., CP and RPP, it should be noted that with respect to the total polyphenol content they differed significantly because in RPP it was twice as high as in CP (Figure 1). The amount of phenolic acids was seven times higher in RPP than in CP, and the content of flavonols was almost four times higher in RPP than in CP. Considering the content of flavonoids, it was found that it was almost four times higher in RPP than in CP. Also, the amount of anthocyanins in CP was twice as low as in RPP (Figure 1).

**Figure 2:**
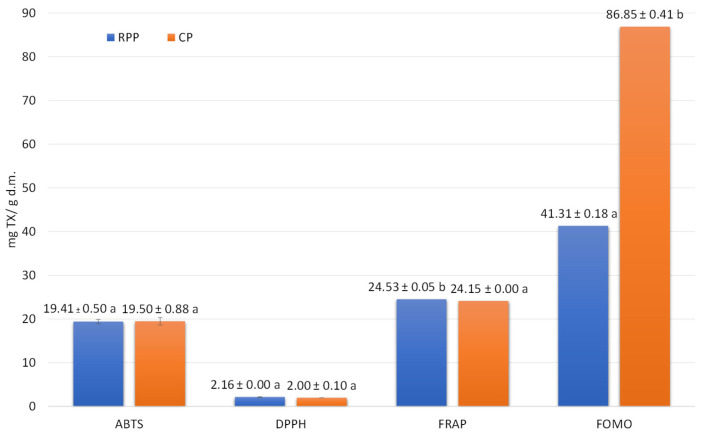
Antioxidant potential of Cherry Pomace (CP) and Red Potato Pulp (RPP). Note: a,b Superscript letters indicate statistically significant differences (α = 0.05).

The antiradical activity measured by ABTS of these two additives was identical (Figure 2).

In the case of determining this activity using DPPH, a slightly higher value was determined in RPP compared to CP. In the case of antioxidant activity determined by the FRAP method, it was slightly lower in CP as compared to RPP; while in CP it was twice as high as in RPP when determining such activity using the FOMO method (Figure 2).

To compare the polyphenol content in this study with that of other authors, it was converted to gallic acid (GA). For RPP, it was around 993 mg GA/100 g dm. According to Kita et al. [17], the total polyphenol content in red and purple-fleshed potatoes was in the range of 250.65–526.25 mg GA/100 g dm, and according to Lachman et al. [18], the content of polyphenols in these potatoes was determined to be in the range of 455–481 mg GA/100 g dm; while in other studies, Kita et al. [19] determined the content of polyphenols in potatoes with red and purple flesh at the level of 226 to 844 mg GA/100 g dm.

Nemś et al. [20] reported the content of total polyphenols in potatoes with colored flesh ranging from 247 to 427 mg GA/100 g dm. It can therefore be stated that the total content of polyphenols in RPP presented in this study was comparable or even higher than the content of these bioactive compounds determined by the above-mentioned authors. According to many authors [21,22], certain discrepancies can result from climatic, soil, and agrotechnical conditions, or potato variety. Other important factors influencing the value of the total polyphenol content in plant material can primarily be the extraction and determination methods of phenolic compounds, and also various ways of expressing the results, for example, a different type of acid or phenolic compound used to calculate polyphenols [23]. In CP, Teslic et al. [15] determined total polyphenols at the level of 140.73 mg of GA/100 g of fresh mass, and in this publication, the amount of polyphenols was 453 mg GA/100 g of dry mass. On the other hand, Okur et al. [24] determined the content of polyphenols in CP within the range of 105–275 mg GA/100 g of fresh weight, which they largely attributed to the extraction of these compounds from plant material.

In the group of potato tuber phenolic compounds, flavonoids occupy an important place. They include many subgroups, namely flavones, flavanones, flavans, flavonols, isoflavones, chalcones, and anthocyanins, constituting a very important element in the health-promoting potential of potato tubers [18,25,26]. In the study by Kita et al. [17], the amount of anthocyanins in potatoes was determined to be in the range of 16.4–57.2 mg of cyanidin-3-glycoside/100 g of dm. In the study by Reyes et al. [21], the content of anthocyanins ranged from 11 to 174 mg of cyanidin-3-glycoside/100 g of dm. Lachman et al. [18] evaluated the content of anthocyanins within 0.7 to 74 mg of cyanidin-3-glycoside/100 g in fresh weight of colored flesh potatoes. In Cherry Pomace, the amount of anthocyanins varied from 12.09 to 174.00 mg of glycoside-3-cyanidin/100 g of fresh weight [24].

It should be noted that the polyphenol profile differed in these two additives (CP and RPP) in terms of quality (Table 1). In the case of RPP, the highest share in the polyphenol content was Pelargonidin 3-(4‴-p-coumaroylrutinoside)-5-glucoside, followed by 3-O-p-Coumaroylquinic acid, 3-O-Caffeoylquinic acid, 5-O-Caffeoylquinic acid. The above-mentioned polyphenols accounted for 81% of the polyphenols identified by the chromatographic method in RPP (Table 1). Also, quercetin derivatives (quercetin 3-O-galactoside, quercetin 3-O-rutinoside and quercetin 3-O-glucoside) had a significant share in the polyphenols identified in RPP (Table 1). The dominant phenolic compounds identified in RPP were 3-O-p-Coumaroylquinic acid, 3-O-Caffeoylquinic acid (neochlorogenic acid), and 5-O-Caffeoylquinic acid (chlorogenic acid–CA) (Table 1). According to many authors [25,27,28,29], phenolic acids constitute a large group of phenolic compounds in the potato tuber, with CA being the dominant one, followed by its derivatives (neo- and cryptochlorogenic acid). According to Rytel et al. [30], potatoes with colored flesh are the richest in chlorogenic and neochlorogenic acid, while the remaining acids are present in slightly smaller amounts. Also, according to Deußer et al. [31], CA and its two above-mentioned isomers are dominant in potatoes with colored flesh, and other acids are present in smaller amounts. Analogous views were presented by Gawlik-Dziki [28] and Mäder et al. [32], who found that potato tubers with different colored flesh are poor in the following phenolic acids: caffeic, coumaric, ferulic, sinapic, and gallic acids. In this work, gallic acid, procyanidin B2, and p-coumaric acid were also determined in small amounts in RPP, which is consistent with the views of some authors [28,30,31,32].

CP was characterized by the highest content of CA (Table 1). It constituted about 46% of the polyphenols identified by the chromatographic method in this material. Cyanidin 3-O-rutinoside constituted 22% of the polyphenols identified in CP (Table 1). In addition, CP also contained polyphenols such as quercetin 3-O-rutinoside-7-O-glucoside, 4-O-caffeoylquinic acid, and kaempferol-3-O-rutinoside. The remaining polyphenols were present in CP in minimal amounts (including gallic acid, procyanidin B1, (+)-catechin, cyanidin 3-O-sophoroside, cyanidin-3-O-glucoside, (−)-epicatechin, 4-O-coumaroylquinic acid, quercetin 3-O-galactoside, quercetin 3-O-galactoside, and 3,5-Di-O-caffeoylquinic acid) (Table 1). CP is also a rich source of anthocyanins, hydroxycinnamic acids (neochlorogenic, chlorogenic, dicaffeoylquinic acids, and p-coumaroylquinic acids) dimer, trimer, tetramer procyanidin, quercetin, and kaempferol, which was confirmed by the literature [15].

It can be stated that the total content of the polyphenols determined by HPLC was definitely higher in the case of CP compared RPP. The dominant role in CP was played by 5-O-Caffeoylquinic acid and Cyanidin 3-O-rutinoside, and quercetin derivatives constituted only a small share of the total polyphenols. In the case of RPP, pelargonidin 3-(4‴-p-coumaroylrutinoside)-5-glucoside, 3-O-p-coumaroylquinic acid, 3-O-caffeoylquinic acid, and 5-O-caffeoylquinic acid constituted 81%, and then quercetin derivatives followed (Table 1).

It can therefore be suggested that these are very valuable additives due to the high content of bioactive compounds from the polyphenol group, and therefore they can be treated as a valuable addition to enriching pasta.

### 2.2. Effect of CP and RPP on the Content of Polyphenols and Antioxidant Potential of Wheat Pasta

It was found that pasta containing CP and RPP was characterized by a significantly higher content of total polyphenols as compared to the control (Table 2). The amount of total polyphenols in pasta supplemented with the above-mentioned additives was 8 to 24 times higher than in the control (Table 2). However, it was noted that the 10 and 20% addition of CP contributed to a higher content of polyphenols in pasta than the same addition of RPP. In contrast, a 30% addition of CP resulted in a 30% lower content of polyphenols in pasta as compared to pasta with a 30% RPP addition (Table 2). In the case of phenolic acids, it was found that even the lowest 10% share of CP resulted in two times lower content of phenolic acids in pasta, than the same share of RPP. The 20% addition of RPP ensured a 15% higher content of phenolic acids compared to pasta with CP. A 30% share of RPP in pasta contributed to an increase in the content of phenolic acids by 64% as compared to pasta with the same level of CP. The amount of flavonols was significantly higher in pasta with 20% and 30% of RPP than in CP pasta. The exception in this respect was pasta with a 10% share of the above-mentioned additives because the amount of flavonols was twice as high in pasta with a share of CP as compared pasta with a share of RPP (Table 2). Considering the content of flavonoids in pastas with the above-mentioned additives, it was found that RPP addition was more beneficial because it guaranteed a higher content of flavonoids in pastas with a 10%, 20%, and 30% share of this pulp, compared to pastas obtained with CP participation. This increase was 16%, 30%, and 60% in the pastas, respectively. It should also be noted that the content of flavonoids was higher in pastas with the addition of CP or RPP, ranging from 2.5 to 9 times higher than in the control (Table 2). Additionally, it was noted that the amount of anthocyanins in pasta with CP addition was lower in comparison to RPP. The presence of anthocyanins was observed in pasta with CP and RPP, but not in the control sample (Table 2).

In Panza et al.’s [33] study on the enrichment of pasta with fig peels (in the amount of 10 to 16%), an increase in the polyphenols content was observed in the range of 46%–84% in fortified pasta as compared to the control. In addition, the content of flavonoids also increased by 13% (with a 10% share of fig peels), but a larger share of this addition caused a decrease in flavonoids compared to the control. In the study by Tolve et al. [34] concerning the influence of the addition of grape pomace on durum pasta properties, it was observed that the 5% and 10% grape pomace addition resulted in a polyphenols increase, respectively, of seven- and twelve-fold as compared to the control. In the study concerning pasta fortified with artichoke bracts and tomato powders [35], it was observed that the addition of bracts increased the content of polyphenols in the range of 21 to 66%, and the addition of tomato powders increased the content of polyphenols in the range of 42 to 80% compared to the control pasta. In a study related to the application of fruit and vegetable by-products as a source of bioactive compounds to preserve pasta [33], it was found that the addition of 3% to 8% of pomegranate peels, broccoli, and olive oil by-products resulted in an increase in the content of both polyphenols and flavonoids in the resulting pasta as compared to the control sample. The content of polyphenols in pasta with a 3% to 8% addition of broccoli by-products increased in the range of 18%–41% as compared to the reference sample. In the case of pomegranate peels, the amount of polyphenols increased by 88% when they were added in the amount of 3% to pasta in comparison to the control. However, with the 8% addition of pomegranate peels to pasta, the content of polyphenols increased three-fold compared to the control. In the case of olive oil by-products, regardless of the amount (3% and 5%), the content of polyphenols oscillated around 0.29 mg of GA/g dm, i.e., it was 70% higher compared to the reference. An 8% addition of olive oil by-products significantly increased the content of polyphenols in pasta by almost twice compared to the control. In the case of flavonoids, their amount increased in pasta with broccoli by-products, from 21% to 3.35 times compared to the standard pasta. The addition of 3% to 8% pomegranate peels caused a 2- to 2.4-fold increase in the amount of flavonoids in pasta. On the other hand, the addition of olive oil by-products from 3% to 8% to pasta contributed to an increase in the flavonoid content in these fortified pasta samples from 2.4 to 3.35 times compared to the control. Additionally, Ajila et al. [36] reported a 3.9-fold increase in the total polyphenol content in pasta with mango peel powder in the range of up to 20%.

Taking into account the quantitative and qualitative profile of polyphenols determined by HPLC, it can be concluded that the control pasta made from wheat flour only is a high-starch product with a low content of bioactive compounds from the polyphenol group. There were only two polyphenolic compounds present: dipcoumaroylspermidine and feruloylquinic acid (Table 3). Only by adding enriching additives, such as CP or RPP, did several bioactive compounds from the polyphenol group (both phenolic acids and flavonoids), along with anthocyanins, appear in the pasta.

Nevertheless, this increase, especially in the case of RPP applied at the level of 10% and 20% into pasta, was smaller than expected. In the case of a 30% RPP addition into pasta, it could be considered as adequate (Table 3). A smaller increase in polyphenol compounds in fortified pasta is related to several factors. It could be suggested that during low-temperature extrusion, some polyphenolic compounds, especially acids, were released from the fiber fraction. Unfortunately, during drying, some polyphenolic compounds (e.g., phenolic acids) can undergo decarboxylation to 4-vinyloguaiacol. According to Michalska et al. [37], polyphenol losses can be quite significant depending on the drying process parameters. Additional losses can be related to cooking, where compound degradation, washing out, and dissolution in water can occur [38]. Jakobek [39] showed that proteins and polyphenols can create covalent bonds, leading to an irreversible linkage and making them more resistant to cooking processes.

In the case of pasta with a share of CP, it was established that although CP itself was rich in various types of polyphenolic compounds, they were lost during the pasta production process. When CP was added to pasta, epigallocatechin, catechin, Cyanidin 3-O-sophoroside, Cyanidin-3-O-glucoside, epigallocatechin, Quercetin 3-O-galactoside, and Quercetin 3-O-glucoside disappeared completely. However, an increase in some acids, e.g., gallic acid, was found in pasta with CP above the level of this additive (Table 3). This was related to the thermal degradation of quercetin derivatives, which can be transformed into phenolic acids as a result of the pasta’s thermal treatment. It can therefore be said that in the case of fruit pomace added to pasta, the additive enriches it with chlorogenic acid and cyanidin. However, this increase was not adequate for the CP level. This increase was smaller, and in the case of gallic acid and procyanidin B1, the amount of these compounds in pasta with pomace was higher than would result from the level of the additive itself (Table 3).

It should also be suggested that cyanidin-3-O-rutinoside exhibited significantly lower stability in the CP-30 sample, as its content reached only 17.51 mg/100 g d.m., indicating substantial degradation during processing. In contrast, chlorogenic acid showed clearly higher retention, with its concentration in CP-30 reaching 37.64 mg/100 g d.m. This distribution of values suggests that the degradation kinetics of cyanidin-3-O-rutinoside were more dynamic than those of chlorogenic acid. This is likely due to the greater structural sensitivity of anthocyanins (classified as flavonoids) to technological factors such as temperature, pH, and oxygen exposure, in comparison with the more stable phenolic acids [37].

Generally, comparing the bioactive compounds that were introduced by CP or RPP to pasta, it can be concluded that RPP turned out to be a much better enriching additive because pasta with this pulp was characterized by a broader range of bioactive compounds from the polyphenol group. However, in the case of CP, it should be noted that this additive contributed to the increase in the content of polyphenolic compounds, but a lot of these compounds were unstable and degraded during pasta preparation. It is consistent with the literature since many authors [37,38,39] believe that the individual stages of pasta preparation contribute to significant losses of polyphenolic compounds. However, the content of gallic and CA in pasta with CP was significantly higher than for RPP pasta. Therefore, it can be said that both RPP and CPP contributed to the increase in the health-promoting value of pasta, adding bioactive compounds that were not present in the control.

It is worth noting that pasta with a share of CP contained a greater amount of gallic acid, a significant amount of chlorogenic acid, and cyanidins (anthocyanins), while pasta with RPP contained pelargonidin. Moreover, pasta enriched with CP was valuable due to the high presence of chlorogenic acid. It has a preventive effect against degenerative diseases and coronary diseases, shows anti-carcinogenic, antiviral, and antibacterial properties, as well as lowers blood pressure [40]. Anthocyanins are also important compounds, i.e., pelargonidin in pasta with RPP and cyanidin in pasta with a share of CP (Table 3). They have anti-inflammatory, antiviral, and antibacterial effects [41]. Anthocyanins reduce the risk of carcinogenic, coronary, and viral diseases, as well as protect against Alzheimer’s disease and diabetes. In addition, anthocyanins improve night vision, reducing the risk of “night blindness” or cataracts [42].

The content of various polyphenolic compounds in pasta with the addition of CP and RPP influenced antioxidant activity (Table 4). However, in the case of DPPH, this antioxidant activity was the same, both for CP- and RPP-enriched pasta as well as for the control. Nevertheless, in the case of the DPPH assay, there is an interference of compounds, especially carotenoids [26], which are present in red potatoes and absorb light at the same wavelength as this assay [43]. Therefore, the results of this analysis were not very clear compared with other analyses. Differences in antioxidant activity were determined using the ABTS method, clearly indicating that the antioxidant activity of the control sample was lower than in enriched pasta. The antioxidant activity of pasta enriched with CP or RPP was higher than the control from 15% to 2.5 times in the case of FRAP and FOMO antioxidant activity; CP- and RPP-enriched pasta samples were also substantially higher than for the reference, with better effects provided by the CP addition. A similar trend was also observed in the case of antioxidant activity determined by the FOMO method, where it was determined that pasta with a share of CP showed 4 to 10 times higher antioxidant activity as compared to the control. In the case of pasta with RPP, the increase in antioxidant activity measured by the FOMO method was two-fold with 10% RPP addition and 2.5 times with 30% RPP addition compared to the control (Table 4).

It should be remembered that antioxidant and antiradical activities are largely determined by the content of individual polyphenols, their chemical structure, in particular, the type, number, and location of substituents in the molecule and the concentration of polyphenolic compounds and their combination with, for example, polysaccharides [18,21,27,44]. According to some authors [18,27,45] chlorogenic, gallic, caffeic, neo, and cryptochlorogenic acids, are responsible for the creation of antioxidant activity and anthocyanins have a synergistic effect with the above-mentioned phenolic acids [46]. It can therefore be said that the higher antiradical and antioxidant activities determined by the ABTS, FRAP, and FOMO methods of pasta with a share of CP compared to pasta with a share of RPP are determined by the many times higher content of gallic and chlorogenic acids in CP-supplemented pasta (Table 2 and Table 4). Strong correlation between antioxidant potential and content of CA and GA were confirmed by high values of Pearson’s correlation coefficients (r), namely CA vs: DPPH (r = 0.978), FRAP (r = 0.972), FOMO (r = 0.966), ABTS (r = 0.848), and GA vs: DPPH (r = 0.912), FRAP (r = 0.999), FOMO (r = 0.987), and ABTS (r = 0.789). The remaining polyphenolic compounds that were present in both RPP- and CP-fortified pasta have a synergistic effect on gallic and chlorogenic acids.

It can therefore be said that in order to increase the health benefits of pasta, it should be fortified with CP and RPP, because both CP and RPP will increase the antioxidant activity and the quantity and quality of polyphenols in the pasta as compared to the control sample.

### 2.3. Effect of CP and RPP on the Content of Phytosterols in Wheat Pasta

Phytosterols have antibacterial, antiviral, anti-inflammatory, and antioxidant properties, reducing the risk of cancer. They also reduce the amount of LDL cholesterol, which of course decreases the risk of heart disease [47]. When considering the content of sterols in pasta, it should be noted that a fairly high content of cholesterol was determined in the control pasta sample, and the addition of RPP caused a decrease in the amount of cholesterol (Table 5). The addition of 20 and 30% CP increased its content in pasta with a share of CP compared to the control. The amount of campesterol in pasta supplemented with both CP and RPP was significantly lower when compared to the control, and these losses were smaller for pasta with a share of CP. The amount of stigmasterol in pasta with 20% RPP was identical to the control. On the other hand, the addition of 10% and 30% RPP to pasta increased stigmasterol level as compared to the control (Table 5). In the case of pasta with addition of CP, the amount of stigmasterol increased from 38% to 250% as compared to the control. On the other hand, the content of beta-sitosterol decreased after the addition of both CP and RPP. This was not surprising because the literature reported that wheat flour was rich in this phytosterol [48]. The remaining two phytosterols contents, i.e., Delta 5-avenasterol and Delta 7-avenosterol, decreased when RPP was added to pasta, and increased, in the range from 11% to 200% for the pasta supplemented with CP, as compared to the control (Table 5).

This is because RPP is rich in beta-sitosterol and stigmasterol, and their amount was significant in RPP pasta. According to Lee et al. [49], among the phytosterols present in RPP, beta-sitosterol and stigmasterol have the largest share. In the case of pasta with a share of CP, the amount of campesterol, stigmasterol, beta-sitosterol, Delta 5-avenasterol, and Delta 7-avenasterol was definitely higher than in RPP-pasta (Table 5). This is related to the fact that CP was characterized by significant amounts of campesterol, Delta 5-avenasterol, Delta 7-avenasterol, beta-sitosterol, and stigmasterol [50], hence their significantly higher content in pasta enriched with CP.

## 3. Materials and Methods

### 3.1. Materials

**Figure 3:**
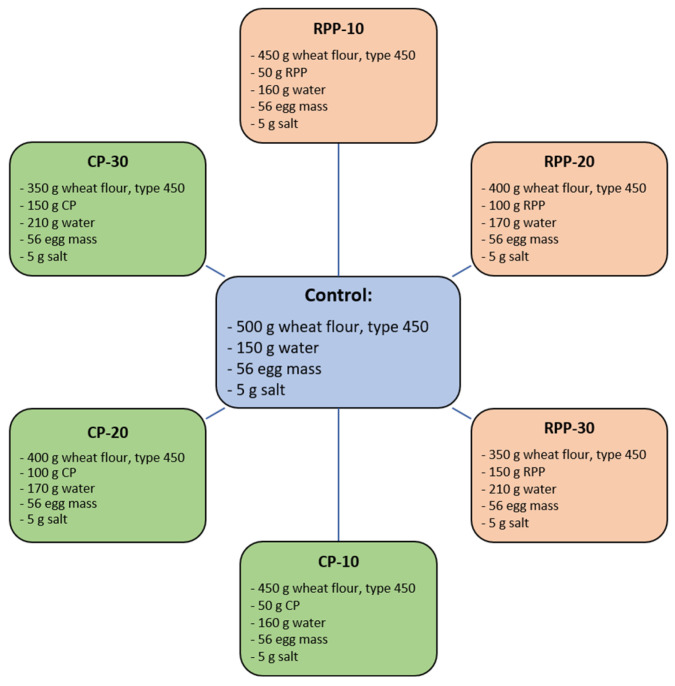
Recipes of the investigated pasta samples.

Both materials and the pasta production procedure were thoroughly described by Gumul et al. [51]. The material for this study was pasta (chifferi rigati and elbows, Figure 3) with different percentages (10%, 20%, and 30%) of freeze-dried RPP or freeze-dried CP (freeze-drying provides the microbiological stability of the material). The CP came from HORTINO Zakład Przetwórstwa Owocowo-Warzywnego Leżajsk Sp. z o.o. (Leżajsk, Poland), while RPP was obtained as a waste material after laboratory isolation procedure of potato starch from a red potato variety Magenta Love (Institute of Environmental Protection and Organic Farming in Spisska Bela, Slovakia, 2021 collection year), according to the procedure of Vischmaan et al. [52].

### 3.2. Pasta Formulation

Pasta enriched with secondary plant-based raw materials—specifically CP and RPP at levels of 10%, 20%, and 30%—was prepared by mixing the ingredients (flour, water, eggs, salt, and the plant-based additive; see Figure 3) using a laboratory spiral mixer SP 12 (Diosna, Osnabrück, Germany) for 15 min at low speed. The dough was then processed using a Gina low-pressure extruder (Ostoni, Cormano, Italy), equipped with a 30 cm-long screw of 5.5 cm diameter and a forming nozzle with a 1.7 mm diameter. Extrusion was carried out at a pressure of approximately 3.4 × 10^5^ Pa and a temperature of 50 °C. The extruded pasta was subsequently dried in a single layer for 30 h at 40 °C in a chamber air-flow dryer, until it reached a moisture content of approximately 12.5%.

### 3.3. Pasta Preparationn

Approximately 110 g of the dried pasta was cooked in 1000 mL of distilled water for a maximum time of 8 min. After cooling, the sample was frozen at −20 °C and lyophilized for 24 h using a Labconco FreeZone 6 freeze-dryer at −47 °C under a pressure of 37 Pa. The freeze-dried pasta was stored at room temperature and ground with a Labconco 3100 grinder prior to further analysis.

### 3.4. Analytical Methods

Antioxidant constituents, antiradical, and antioxidant activities were determined in the methanol–acetone extracts. A total of 1.0 g of the sample was dissolved in 40 cm^3^ 0.16 N HCl and then in 80% methanol, shaken in the dark for 120 min at 23 °C (WB22, Memmert, Schwabach, Germany), and next subjected to ultrasound action for 10 min at 23 °C and centrifuged (15 min, 1050× *g*) in a centrifuge (MPW-350, MED Instruments, Warsaw, Poland). The supernatant was decanted, and residuals were extracted using a 40 cm^3^ of 70% acetone and again shaken in darkness for an additional 120 min at 23 °C (WB22, Memmert, Schwabach, Germany), with ultrasound for 10 min at 23 °C and then centrifuged (15 min, 1050× *g*) in a centrifuge (MPW-350, MED Instruments, Warsaw, Poland). The supernatant was decanted, mixed with methanol extract, and then stored at −20 °C for further analyses.

Determination of total polyphenols content (TPC) was performed without using Folin–Ciocalteu reagent, according to Mazza et al. [53], with the modification by Oomah et al. [54]. The content of phenolic acids was measured using a spectrophotometrical method, according to the abovementioned methods, as same as the content of flavonols and anthocyanins. The content of flavonoids was evaluated using a spectrophotometrical method, according to El Hariri et al. [55].

Antiradical and antioxidant activity was evaluated with the application of popular methods (DPPH, ABTS, FOMO and FRAP). Antiradical activity was measured by spectrophotometric method, using DPPH according to Brand-Williams et al. [56] and ABTS according to Re et al. [57]. Antioxidant activity was measured spectrophotometrically using a phosphomolybdenum complex FOMO method according to Prieto et al. [58] and using the FRAP method [59].

### 3.5. Phytosterols in Food Determination Using Gas Chromatography

The determination of phytosterols in food using gas chromatography was conducted following the methodologies described by Hussain et al. [60], Oracz et al. [61], and Zhang et al. [62]. Sample preparation involved weighing 0.2 g (±0.0001 g) of the material into a 20 mL vial. To each vial, 4 cm^3^ of freshly prepared saponification reagent—comprising 3.9 cm^3^ of 2 M potassium hydroxide (KOH) in methanol and 0.5 mL of 10% ascorbic acid—was added. The vials were sealed and incubated at 85 °C for 40 min, followed by cooling to room temperature.

The reaction mixture was then transferred to 30 mL centrifuge tubes containing 10 cm^3^ of hexane and 10 cm^3^ of saturated sodium chloride (NaCl) solution. The tubes were tightly closed, shaken for 10 min at 175 rpm, and centrifuged at 6000 rpm for 10 min. The upper hexane layer was carefully collected, transferred into 20 mL vials, and evaporated under a nitrogen stream. The residue was then reconstituted with 1 cm^3^ of hexane and sonicated for 10 s.

Prior to analysis, the samples were filtered using a nylon syringe filter with a pore size of 0.2–0.45 µm (ProSource Scientific, Calgary, AB, Canada). Gas chromatographic analysis was carried out using a Shimadzu GC 2010 Plus gas chromatograph equipped with a flame ionization detector (FID) (Shimadzu Corp., Kyoto, Japan).

### 3.6. Phenolic Compound Analysis by UHPLC–DAD–ESI–MS/MS

Phenolic compounds present in the extracts obtained from both the pasta samples and the incorporated plant-based additives (Cherry Pomace—CP and Red Potato Pulp—RPP) were determined using the UHPLC–DAD–ESI–MS/MS technique. The analytical procedure was adapted, with slight modifications, from the method previously described in detail by Oracz et al. [63]. The characterization encompassed samples of the enriched pasta as well as the raw plant-based components.

Chromatographic analysis was carried out using a Dionex UltiMate 3000 UHPLC+ system (Thermo Fisher Scientific Inc., Waltham, MA, USA), equipped with a diode array detector (DAD). For mass spectrometry, a Transcend TLX-2 LC system coupled with a Q-Exactive Orbitrap mass spectrometer (Thermo Scientific, Hudson, NH, USA) was employed, operating with a heated electrospray ionization source (HESI–II).

Phenolic compounds were separated on an Accucore C18 column (2.1 × 150 mm, 2.6 µm particle size), thermostated at 30 °C. The mobile phase consisted of 0.1% formic acid in water (solvent A) and acetonitrile (solvent B), with the following gradient profile at a flow rate of 0.35 mL/min: 0–8 min, 1–5% B; 8–15 min, 5–8% B; 15–20 min, 8–10% B; 20–25 min, 10–15% B; 25–35 min, 15–20% B; 35–40 min, 20–25% B; 40–50 min, 25–90% B; 50–53 min, 90% B; 53–58 min, 90–1% B. The system was re-equilibrated for 7 min before the next injection.

Detection was performed at 280 nm for hydroxybenzoic acids and their derivatives, and at 320 nm for hydroxycinnamic acids and their derivatives. Mass spectrometric detection was operated in negative ion mode with a scan range of *m*/*z* 100–1500. Instrument settings included a capillary voltage of 4500 V, capillary temperature of 275 °C, heater gas temperature of 320 °C, and nitrogen as the sheath (35 units) and auxiliary gas (15 units). MS/MS spectra were acquired via collision-induced dissociation (CID) with normalized collision energy (NCE) set at 20%.

Compound identification was based on retention times, UV–Vis spectra, full scan mass spectra, and MS/MS fragmentation profiles, compared with authentic standards and the available literature. Quantitative analysis followed the external standard method. Calibration curves were prepared in triplicate using standard solutions at concentrations ranging from 0.01 to 100 mg/L. Linearity was verified using regression analysis, and correlation coefficients were determined. Limits of detection (LOD) and quantification (LOQ) were calculated as signal-to-noise ratios of ≥3 and ≥10, respectively.

Quantified compounds included gallic acid, protocatechuic acid, ellagic acid, vanillic acid, p-hydroxybenzoic acid, syringic acid, caffeic acid, ferulic acid, p-coumaric acid, chlorogenic acid, sinapic acid, 3,4-di-O-caffeoylquinic acid, 2,5-dihydroxybenzoic acid, quercetin 3-O-galactoside, quercetin 3-O-glucoside, and quercetin 3-O-rutinoside (rutin). The results were expressed as milligrams of compound per 100 g of dry matter (mg/100 g d.m.).

### 3.7. Statistical Analysis

The experimental data were subjected to analysis of variance (Duncan’s test), at the confidence level of 0.05, by means of Statistica v. 8.0 (Statsoft, Inc., Tulsa, OK, USA). All measurements were performed at least in duplicate. Results are presented as means with standard deviation. Additionally, Pearson correlation coefficients ^®^ were calculated using Microsoft Excel for Microsoft 365 (Version 2403), based on selected data series.

## 4. Conclusions

It was found that both CP and RPP were sources of bioactive compounds from the polyphenol group. The amount of polyphenols determined by HPLC was definitely higher in the case of CP than in RPP, and the dominant role in CP was played by 5-O-Caffeoylquinic acid and Cyanidin 3-O-rutinoside. Quercetin derivatives accounted for only a small share of the total polyphenols. In the case of RPP, Pelargonidin 3-(4‴-p-coumaroylrutinoside)-5-glucoside, then 3-O-p-Coumaroylquinic acid, 3-O-Caffeoylquinic acid, and 5-O-Caffeoylquinic acid accounted for 81%. The rest were quercetin derivatives. It was found that pasta with the addition of CP and RPP was characterized by a higher content of total polyphenols as well as phenolic acids, flavonoids, flavanols, and anthocyanins compared to the control sample. It was also observed that RPP enriched the pasta with a large range of bioactive compounds from the polyphenol group. For pasta with CP, a substantial amount of bioactive compounds from the polyphenol group were unstable during pasta production. The samples of pasta with CP were characterized by very high antioxidant activity (ABTS, FOMO, FRAP) due to a large amount of phenolic acids (gallic and chlorogenic acids) and anthocyanins acting synergistically, in relation to pasta with a share of RPP.

It was also observed that pasta with CP was characterized by a higher content of phytosterols such as campesterol, stigmasterol, beta-sitosterol, Delta-5-avenasterol and Delta-7-avenasterol than pasta with a share of RPP. To sum up, in order to obtain pasta with a high antioxidant potential with a large amount of biologically active compounds (polyphenols and phytosterols), CP should be applied rather than RPP. In particular, a 30% addition of CP to pasta guarantees the above-mentioned health-promoting values of the innovative functional pasta obtained in this way.

## Figures and Tables

**Table 1:** Polyphenols and phytosterols compositions in raw materials.

Compound *	Content (mg/100 g d.m.)	
	CP	RPP
	Polyphenols	
Gallic acid	1.75 ± 0.03 ^b^*	1.32 ± 0.01 ^a^
Procyanidin B1	9.44 ± 0.18 ^b^	4.68 ± 0.04 ^a^
3-O-Caffeoylquinic acid (neochlorogenic acid)	349.65 ± 6.71 ^b^	35.41 ± 0.32 ^a^
(−)-Epigallocatechin	26.44 ± 0.51 ^a^	0.00 ± 0.00
3-O-p-Coumaroylquinic acid	0.00 ± 0.00	59.88 ± 0.54 ^a^
5-O-Caffeoylquinic acid (chlorogenic acid)	1010.78 ± 19.4 ^b^	33.69 ± 0.3 ^a^
(+)-Catechin	9.40 ± 0.18 ^a^	0.00 ± 0.00
Cyanidin 3-O-sophoroside	11.13 ± 0.21 ^a^	0.00 ± 0.00
4-O-Caffeoylquinic acid	116.81 ± 2.24 ^b^	2.72 ± 0.02 ^a^
Cyanidin-3-O-glucoside	11.38 ± 0.22 ^a^	0.00 ± 0.00
(−)-Epicatechin	8.31 ± 0.16 ^a^	0.00 ± 0.00
4-O-coumaroylquinic acid	4.36 ± 0.08 ^a^	0.00 ± 0.00
Procyanidin B2	0.00 ± 0.00	12.77 ± 0.11 ^a^
p-Coumaric acid	0.00 ± 0.00	1.96 ± 0.02 ^a^
Pelargonidin 3-(4‴-p-coumaroylrutinoside)-5-glucoside	0.00 ± 0.00	428.21 ± 3.84 ^a^
Procyanidin C1	0.00 ± 0.00	7.68 ± 0.07 ^a^
Quercetin 3-O-rutinoside-7-O-glucoside	53.00 ± 1.02 ^a^	0.00 ± 0.00
Quercetin 3-O-(2′-glucosyl)-rutinoside	0.00	15.60 ± 0.14 ^a^
Quercetin 3-O-galactoside	5.56 ± 0.11 ^a^	39.84 ± 0.36 ^b^
Quercetin 3-O-rutinoside	8.17 ± 0.16 ^b^	6.39 ± 0.06 ^a^
Cyanidin 3-O-rutinoside	473.25 ± 9.08 ^a^	0.00
Quercetin 3-O-glucoside	2.78 ± 0.05 ^a^	9.66 ± 0.09 ^b^
Quercetin 3-O-sulfate	0.00 ± 0.00	23.0 ± 0.21 ^b^
Kaempferol-3-O-rutinoside	45.82 ± 0.88 ^a^	0.00 ± 0.00
3,5-Di-O-caffeoylquinic acid	6.75 ± 0.13 ^a^	0.00 ± 0.00
Quercetin	0.00 ± 0.00	0.00 ± 0.00
	Phytosterols	
Cholesterol	2.71 ± 0.00 ^a^	0.00 ± 0.00
Stignasterol	1.07 ± 0.12 ^a^	1.01 ± 0.07 ^a^
Sitosterol	17.04 ± 0.27 ^b^	1.53 ± 0.10 ^a^
Campesterol	2.53 ± 0.03 ^a^	0.00 ± 0.00
Δ 5avenasterol	1.52 ± 0.04 ^a^	0.00 ± 0.00
Δ 7avenasterol	0.84 ± 0.11 ^a^	0.00 ± 0.00

* All values in the rows marked with different superscripts are statistically different (α = 0.05); CP—Cherry Pomace, RPP—Red Potato Pulp.

**Table 2:** Polyphenols content in the pasta samples.

	Total Polyphenol Content (mg Catechin/g d.m.)	Phenolic Acids (mg Ferulic Acid/g d.m.)	Flavonols (mg Quercetin/g d.m.)	Anthocyanins (mg Glucoside Cyjanidyn/g d.m.)	Flavonoids (mg Rutin/g d.m.)
Control	13.04 ± 0.40 ^a^*	0.07 ± 0.00 ^a^	0.00 ± 0.00 ^a^	0.00 ± 0.00 ^a^	13.2 ± 0.60 ^a^
CP-10	123.67 ± 1.19 ^c^	5.42 ± 0.13 ^b^	4.10 ± 0.21 ^c^	11.19 ± 0.00 ^c^	29.16 ± 1.22 ^b^
CP-20	203.80 ± 1.47 ^e^	11.22 ± 0.09 ^d^	5.80 ± 0.53 ^d^	24.9 ± 1.14 ^d^	42.70 ± 1.80 ^d^
CP-30	216.08 ± 0.34 ^f^	14.08 ± 0.00 ^e^	8.08 ± 0.16 ^e^	33.73 ± 1.10 ^f^	69.00 ± 1.52 ^f^
RPP-10	109.76 ± 0.00 ^b^	10.25 ± 0.12 ^c^	2.39 ± 0.12 ^b^	8.82 ± 0.50 ^b^	33.90 ± 0.34 ^c^
RPP-20	182.40 ± 1.40 ^d^	13.03 ± 0.64 ^f^	9.79 ± 0.87 ^f^	26.60 ± 1.01 ^e^	57.18 ± 0.61 ^e^
RPP-30	323.93 ± 0.89 ^g^	23.78 ± 0.23 ^g^	19.49 ± 0.34 ^g^	36.16 ± 0.81 ^g^	110.26 ± 1.52 ^g^

* All values in the column marked with different superscripts are statistically different (α = 0.05).

**Table 3:** Polyphenols profile in pasta samples (mg/100 g d.m.).

Compound *	Control	CP-10	CP-20	CP-30	RPP-10	RPP-20	RPP-30
Gallic acid	0.00 ± 0.0 ^a^*	1.65 ± 0.03 ^d^	2.74 ± 0.05 ^e^	4.20 ± 0.08 ^f^	0.18 ± 0 ^b^	0.22 ± 0 ^b^	1.07 ± 0.01 ^c^
ProcyanidinB1	0.00 ± 0.0 ^a^	2.29 ± 0.04 ^b^	3.67 ± 0.07 ^b^	7.88 ± 0.15 ^f^	4.32 ± 0.04 ^c^	4.76 ± 0.04 ^d^	5.94 ± 0.05 ^e^
3-O-Caffeoylquinic acid (neochlorogenic acid)	0.00 ± 0.0 ^a^	0.52 ± 0.01 ^b^	6.08 ± 0.12 ^e^	9.31 ± 0.18 ^f^	1.34 ± 0.01 ^c^	3.81 ± 0.03 ^d^	9.92 ± 0.09 ^g^
(−)-Epigallocatechin	0.00 ± 0.0 ^a^	0 ^a^	0 ^a^	0 ^a^	-	-	-
3-O-p-Coumaroylquinic acid	0.00 ± 0.0 ^a^	-	-	-	2.41 ± 0.02 ^b^	6.55 ± 0.06 ^c^	17.09 ± 0.15 ^d^
5-O-Caffeoylquinic acid (chlorogenic acid)	0.00 ± 0.0 ^a^	4.12 ± 0.08 ^d^	24.6 ± 0.47 ^f^	37.64 ± 0.72 ^g^	1.94 ± 0.02 ^b^	3.79 ± 0.03 ^c^	9.69 ± 0.09 ^e^
(+)-Catechin	0.00 ± 0.0 ^a^	0.00 ± 0.0 ^a^	0.00 ± 0.0 ^a^	0.00 ± 0.0 ^a^	-	-	-
Cyanidin3-O-sophoroside	-	-	-	-	-	-	-
4-O-Caffeoylquinic acid	0.00 ± 0.0 ^a^	0.44 ± 0.01 ^c^	3.86 ± 0.07 ^e^	5.91 ± 0.11 ^f^	1.94 ± 0.02 ^d^	0.27 ± 0 ^b^	0.44 ± 0 ^c^
Cyanidin-3-O-glucoside	0.00 ± 0.0	0	0	0	-	-	-
(−)-Epicatechin	0.00 ± 0.0	0	0	0	-	-	-
4-O-coumaroylquinic acid	0.00 ± 0.0 ^a^	0.03 ± 0 ^b^	0.05 ± 0 ^c^	0.08 ± 0 ^d^	-	-	-
Procyanidin B2	0.00 ± 0.0 ^a^	-	-	-	1.04 ± 0.01 ^b^	3.44 ± 0.03 ^c^	9.34 ± 0.08 ^d^
p-Coumaric acid	0.00 ± 0.0 ^a^	-	-	-	0.14 ± 0 ^b^	0.47 ± 0 ^c^	1.27 ± 0.01 ^d^
Pelargonidin3-(4‴-p-coumaroylrutinoside)-5-glucoside	0.00 ± 0.0 ^a^	-	-	-	11.80 ± 0.11 ^b^	67.16 ± 0.6 ^c^	189.52 ± 1.7 ^d^
Procyanidin C1	0.00 ± 0.0 ^a^	-	-	-	0.51 ± 0 ^a^	1.31 ± 0.01 ^b^	4.23 ± 0.04 ^d^
Quercetin3-O-rutinoside-7-O-glucoside	0.00 ± 0.0 ^a^	0.97 ± 0.02 ^a^	2.94 ± 0.06 ^b^	4.5 ± 0.09 ^c^	-	-	-
Quercetin3-O-(2′-glucosyl)-rutinoside	0.00 ± 0.0 ^a^	-	-	-	0.77 ± 0.01 ^a^	2.11 ± 0.02 ^b^	4.96 ± 0.04 ^c^
Quercetin3-O-galactoside	0.00 ± 0.0 ^a^	0 ^a^	0 ^a^	0 ^a^	4.74 ± 0.04 ^b^	10.43 ± 0.09 ^c^	23.55 ± 0.21 ^d^
Quercetin3-O-rutinoside	0.00 ± 0.0 ^a^	1.44 ± 0.03 ^b^	2.88 ± 0.06 ^d^	4.32 ± 0.08 ^e^	2.37 ± 0.02 ^c^	5.22 ± 0.05 ^f^	11.77 ± 0.11 ^g^
Cyanidin3-O-rutinoside	0.00 ± 0.0 ^a^	3.54 ± 0.07 ^b^	7.06 ± 0.14 ^c^	17.51 ± 0.34 ^d^	-	-	-
Quercetin3-O-glucoside	0.00 ± 0.0 ^a^	0 ^a^	0 ^a^	0 ^a^	0.77 ± 0.01 ^a^	1.29 ± 0.01 ^b^	3.04 ± 0.03 ^c^
Quercetin3-O-sulfate	0.00 ± 0.0 ^a^	-	-	-	1.25 ± 0.01 ^a^	2.95 ± 0.03 ^b^	6.86 ± 0.06 ^c^
Kaempferol-3-O-rutinoside	0.00 ± 0.0 ^a^	3.81 ± 0.07 ^b^	10.35 ± 0.2 ^c^	16.47 ± 0.32 ^d^	-	-	-
3,5-Di-O-caffeoylquinic acid	0.00 ± 0.0 ^a^	0.14 ± 0 ^a^	0.61 ± 0.01 ^b^	0.94 ± 0.02 ^c^	-	-	-
Quercetin	0.00 ± 0.0 ^a^	0.14 ± 0 ^a^	0.61 ± 0.01 ^b^	0.92 ± 0.02 ^c^	-	-	-
Dipcoumaroylspermidine	0.35 ± 0.01 ^d^	0.21 ± 0.03 ^c^	0.16 ± 0.02 ^c^	0 ± 0 ^a^	0.24 ± 0.02 ^c^	0.13 ± 0 ^b^	0 ± 0 ^a^
Feruloylquinic acid	0.10 ± 0.00 ^d^	0.07 ± 0.01 ^c^	0 ± 0 ^a^	0 ± 0 ^a^	0.05 ± 0 ^b^	0 ± 0 ^a^	0 ± 0 ^a^

* All values in the rows marked with different superscripts are statistically different (α = 0.05); Control—pasta with no additions, CP-10—pasta with a 10% share of Cherry Pomace, CP-20—pasta with a 20% share of Cherry Pomace, CP-30—pasta with a 30% share of Cherry Pomace, RPP-10—pasta with a 10% share of Red Potato Pulp, RPP-20—pasta with a 20% share of Red Potato Pulp, RPP-30—pasta with a 30% share of Red Potato Pulp.

**Table 4:** Antioxidative activities (mg TX/g d.m.) of pasta samples.

	DPPH	FRAP	FOMO	ABTS
Control	0.61 ± 0.01 ^a^*	0.06 ± 0.00 ^a^	2.57 ± 0.15 ^a^	6.36 ± 0.53 ^a^
CP-10	0.64 ± 0.07 ^a^	2.10 ± 0.42 ^b^	8.88 ± 0.31 ^d^	9.45 ± 0.21 ^c^
CP-20	0.85 ± 0.10 ^a^	4.60 ± 0.40 ^c^	16.75 ± 0.28 ^e^	14.15 ± 0.13 ^f^
CP-30	1.02 ± 0.15 ^a^	6.89 ± 0.36 ^d^	21.79 ± 0.80 ^f^	15.89 ± 0.24 ^g^
RPP-10	0.62 ± 0.00 ^a^	0.43 ± 0.00 ^a^	4.58 ± 0.62 ^bc^	7.63 ± 0.19 ^b^
RPP-20	0.68 ± 0.04 ^a^	0.50 ± 0.00 ^a^	3.76 ± 0.07 ^ab^	11.88 ± 0.00 ^d^
RPP-30	0.78 ± 0.09 ^a^	1.11 ± 0.11 ^ab^	5.97 ± 0.01 ^c^	13.45 ± 0.35 ^e^

* All values in the columns marked with different superscripts are statistically different (α = 0.05).

**Table 5:** Phytosterols profile in the pasta samples.

	Cholesterol (mg/100 g)	Campesterol (mg/100 g)	Stigmasterol (mg/100 g)	β-Sitosterol (mg/100 g)	Δ-5-Avenasterol (mg/100 g)	Δ-7-Avenasterol (mg/100 g)
Control	53.85 ± 1.60 ^d^*	3.04 ± 0.09 ^d^	0.26 ± 0.00 ^a^	13.15 ± 0.37 ^d^	0.35 ± 0.00 ^b^	0.54 ± 0.03 ^c^
CP-10	34.59 ± 0.02 ^a^	1.67 ± 0.01 ^ab^	0.36 ± 0.19 ^b^	7.8 ± 0.03 ^b^	0.39 ± 0.01 ^c^	0.76 ± 0.01 ^e^
CP-20	51.71 ± 1.22 ^d^	2.46 ± 0.05 ^c^	0.56 ± 0.00 ^c^	11.39 ± 0.26 ^c^	0.48 ± 0.04 ^d^	0.62 ± 0.02 ^d^
CP-30	73.49 ± 0.75 ^e^	2.48 ± 0.05 ^c^	0.63 ± 0.01 ^d^	10.79 ± 0.23 ^c^	0.69 ± 0.00 ^e^	0.80 ± 0.02 ^f^
RPP-10	39.99 ± 0.27 ^b^	1.79 ± 0.01 ^b^	0.36 ± 0.01 ^b^	7.82 ± 0.06 ^b^	0.18 ± 0.01 ^a^	0.21 ± 0.04 ^a^
RPP-20	34.21 ± 0.12 ^a^	1.60 ± 0.01 ^a^	0.24 ± 0.04 ^a^	6.57 ± 0.01 ^a^	0.17 ± 0.01 ^a^	0.23 ± 0.01 ^a^
RPP-30	45.46 ± 0.19 ^c^	1.69 ± 0.02 ^ab^	0.32 ± 0.01 ^b^	7.31 ± 0.00 ^ab^	0.17 ± 0.01 ^a^	0.32 ± 0.04 ^b^

* All values in the columns marked with different superscripts are statistically different (α = 0.05).

## Data Availability

Data is contained within the article.

## Abbreviations

The following abbreviations are used in this manuscript:

CP	Cherry Pomace
RPP	Red Potato Pulp
Control	pasta with no additions
CP-10	pasta with a 10% share of Cherry Pomace
CP-20	pasta with a 20% share of Cherry Pomace
CP-30	pasta with a 30% share of Cherry Pomace
RPP-10	pasta with a 10% share of Red Potato Pulp
RPP-20	pasta with a 20% share of Red Potato Pulp
RPP-30	pasta with a 30% share of Red Potato Pulp
